# Genome dynamics and evolution in yeasts: A long-term yeast-bacteria competition experiment

**DOI:** 10.1371/journal.pone.0194911

**Published:** 2018-04-06

**Authors:** Nerve Zhou, Michael Katz, Wolfgang Knecht, Concetta Compagno, Jure Piškur

**Affiliations:** 1 Department of Biology, Lund University, Lund, Sweden; 2 Department of Biological Sciences and Biotechnology, Botswana International University of Science and Technology, Private Bag 16, Palapye, Botswana; 3 Carlsberg Laboratories, Gamle Carlsberg Vej 10, Copenhagen V, Denmark; 4 Lund Protein Production Platform, Lund University, Lund, Sweden; 5 Department of Food, Environmental and Nutritional Sciences, University of Milan, Milan, Italy; Leibniz Institut - Deutsche Sammlung von Mikroorganismen und Zellkulturen GmbH, GERMANY

## Abstract

There is an enormous genetic diversity evident in modern yeasts, but our understanding of the ecological basis of such diversifications in nature remains at best fragmented so far. Here we report a long-term experiment mimicking a primordial competitive environment, in which yeast and bacteria co-exist and compete against each other. Eighteen yeasts covering a wide phylogenetic background spanning approximately 250 million years of evolutionary history were used to establish independent evolution lines for at most 130 passages. Our collection of hundreds of modified strains generated through such a rare two-species cross-kingdom competition experiment re-created the appearance of large-scale genomic rearrangements and altered phenotypes important in the diversification history of yeasts. At the same time, the methodology employed in this evolutionary study would also be a non-gene-technological method of reprogramming yeast genomes and then selecting yeast strains with desired traits. Cross-kingdom competition may therefore be a method of significant value to generate industrially useful yeast strains with new metabolic traits.

## Introduction

The enormous genetic variation evident in modern yeasts may provide a simple explanation of their adaptations to a wide range of niches. As much as we understand the molecular mechanisms of yeast evolutionary history, our understanding of the ecological basis of such adaptive mechanisms remains so far incomplete. Adaptive evolution experiments entailing the application of a selective pressure, for example, environmental stresses caused by nutrient limitation and or inhibitory substances, have often been used to explain changes in the genomes of natural populations [1–5]. A number of diverse genetic mechanisms and their phenotypes have been reconstructed and described using *Escherichia coli* and *Saccharomyces cerevisiae*. Among them are the fixation of beneficial mutations [6], genetic hitchhiking [7], and physiological heterogeneity in experimentally evolved yeast populations [8]. In addition, the strategic collective physiology of niche defending [9], ecological and genome dynamics [10], stable phenotypes borne of clonal interference [11, 12] and trade-offs behind observed laboratory adaptations [13] have been described. Long-term experimental evolution experiments studies revealed that genomic rearrangements underlie increased fitness and adaptation in *S*. *cerevisiae* [11, 14]. Other distant relatives of the baker´s yeasts have also been reported to respond to environmental perturbations by genome restructuring [15, 16].

Although such experimental evolution experiments have shed light on outcomes due to natural selection, most experiments have relied on single-species in isolation often not taking into account the community context of the natural world as emphasized already by Darwin in the *Origin of Species* [17]. Most ecological niches, thought to be the most predominant natural niches for yeasts, e.g. ripening fruits, are in fact characterized by a complex interaction of a multitude of other co-habitants from across the kingdoms, such as bacteria [18, 19]. The significance of the inter-species interactions on the tempo of genome evolution in yeasts in a laboratory set up has not yet been fully explored.

Bacteria are potential “hurdles” that could influence the evolution of adaptive life strategies in yeast by rewiring of their genetic networks; for example, carbon metabolism pathways and regulatory networks [16, 20]. They secrete antifungal compounds such as chitinases [21, 22], central carbon metabolism-impairing compounds such as weak organic acids, prions and long-chain fatty acids [23–29]. On the other hand, yeasts that accumulate microbial stressors such as ethanol and acetic acid [30–32], volatile antimicrobial substances [33, 34] and other potent anti-bacterial substances [35] may have evolved a counter strategy to outcompete bacteria and probably other taxa. How microbes behave as weeds in a community setup where the battle for dominance is based on aggressive phenotypes, tolerance to potent stressors, as well as on the ability to store and utilize limiting resources has been comprehensively reviewed in [35].

In our study, we explored whether we could reconstruct molecular mechanisms that have led to diversity in modern yeast genomes. Using a yeast-bacteria competition as a probable trigger of genetic diversity in natural environments, we set up a laboratory evolution experiment to study its outcomes. Eighteen yeasts from the family *Saccharomycetaceae* covering a wide phylogenetic background spanning over 250 million years of descent [36] were kept in the presence of competitors for at most 130 passages. Karyotyping of several samples taken along the timeline of the experiment revealed that eight out of the 18 yeasts underwent large-scale genomic rearrangements. This highlights the role played by cross-kingdom competition in nature. High-throughput phenotypic characterization of these evolved strains further revealed the biological changes underlying the observed genomic reorganizations. Our results suggest that interspecific interactions between yeasts and bacteria in nature may have accelerated genome evolution and influenced phenotypic diversification characteristics of the modern yeasts.

## Materials and methods

### Strains used in this study

Eighteen yeasts from the family *Saccharomycetaceae* covering a wide phylogenetic background of evolutionary history were used to establish independent evolution lines (Table 1). A list of bacteria, isolated from sugar rich niches, used to compete with the yeasts is found in Table 1. It is noteworthy that *Pseudomonas fluorescens* strain isolated in Lund, Sweden, was previously identified as *Pseudomonas fluorescens*. subsp *cellulosa* (NCIMB 10 462) [37] but has been reclasssified as *Cellvibrio japonicus* sp. *nov* [38].

**Table 1:** A list of strains used in this study.

Origin	Lund culture collection number	Species name
**Yeasts**		
CBS 8340	Y706	*Saccharomyces cerevisiae* A1
CBS 7413	Y1714	*Saccharomyces cerevisiae* A2
CBS 12357	Y1693	*Saccharomyces eubayanus*
CBS 138	Y475	*Candida glabrata*
CBS 2926	Y1055	*Torulaspora pretoriensis*
CBS 6340	Y688	*Lachancea thermotolerans*
CBS 3082	Y057	*Lachancea kluyveri*
CBS 8778	Y1057	*Kluyveromyces nonfermentans*
CBS 2745	Y113	*Kluyveromyces wickerhamii*
CBS 2359	Y707	*Kluyveromyces lactis* A1
CBS 2359	Y1376	*Kluyveromyces lactis* A2
CBS 712	Y1058	*Kluyveromyces marxianus*
CBS 109.51	Y1001	*Eremothecium gossypii*
CBS 6920	Y1399	*Debaryomyces hanseni*
CBS 2499	Y879	*Dekkera bruxellensis*
CBS 77	Y863	*Dekkera anomala*
CBS 6116	Y919	*Brettanomyces naardenensis*
CBS 4805	Y893	*Brettanomyces custersianus*
**Bacteria**		
Eh318 (CUCPB 2140)	P1068	*Pantoea agglomerans*
AS9 (CCUG 61396)	P1070	*Serratia plymuthica*
PS216 (BGSC 3A36)	P1081	*Bacillus subtilis*
ATCC^®^ 10712 ^TM^ (NRRL B-2277)	P1071	*Streptomyces venezuelae*
NCDO 2118	P1069	*Lactococcus lactis* subsp. *lactis*
NCIMB 10462	P1076	*Pseudomonas fluorescens*

CBS, *Centraalbureau voor Schimmelcultures* (or CBS-KNAW Westerdijk Fungal Biodiversity Institute); CUCPB, Cornell University Collection of Phytopathogenic Bacteria; CCUG, Culture Collection, University of Gothenburg; BGSC, Bacillus Genetic Stock Centre; ATCC, American Type Culture Collections; NCDO, now National Collection of Food Bacteria; NCFB: National Collection of Food Bacteria. All strains are availbale from the Jure Piskur´s Group culture collections at Lund University, Sweden.

### Experimental evolution strategy

**Fig 1:**
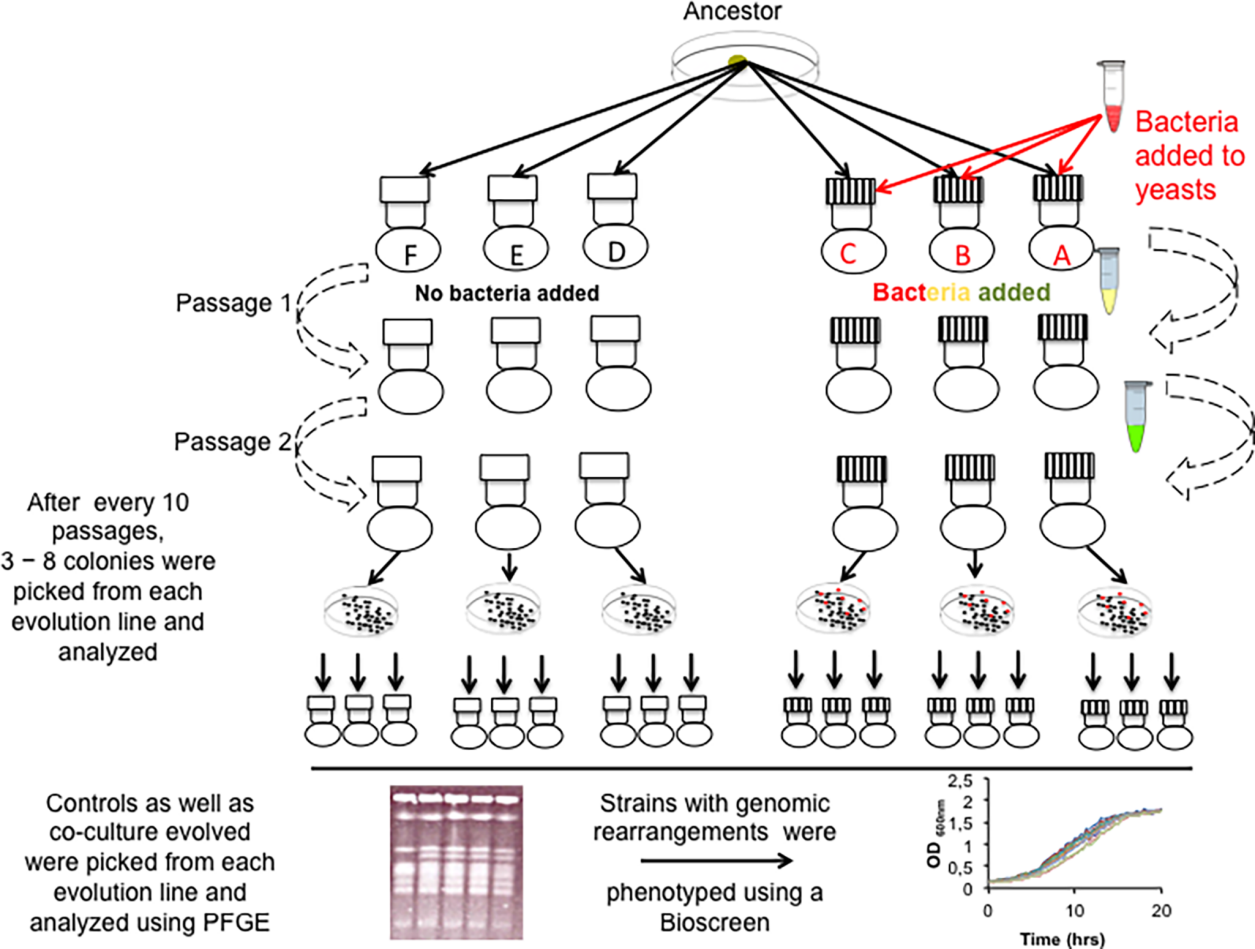
Graphic view of the experimental evolution setup. Three flasks labeled A, B, and C in red (striated caps) were inoculated with both yeast and bacteria whereas the other three flasks labeled D, E, and F in black (caps with no fill) were inoculated with yeast only (controls). The cultures were successively passaged for at most passages depending on the strain (see Table 2). After every 10 passages, cultures were withdrawn and karyotyped using pulse field gel electrophoresis (PFGE). Those that underwent genomic rearrangements were then phenotyped using a high-throughput micro-cultivation instrument, Bioscreen C (Oy Growth Curves, Finland).

**Fig 2:**
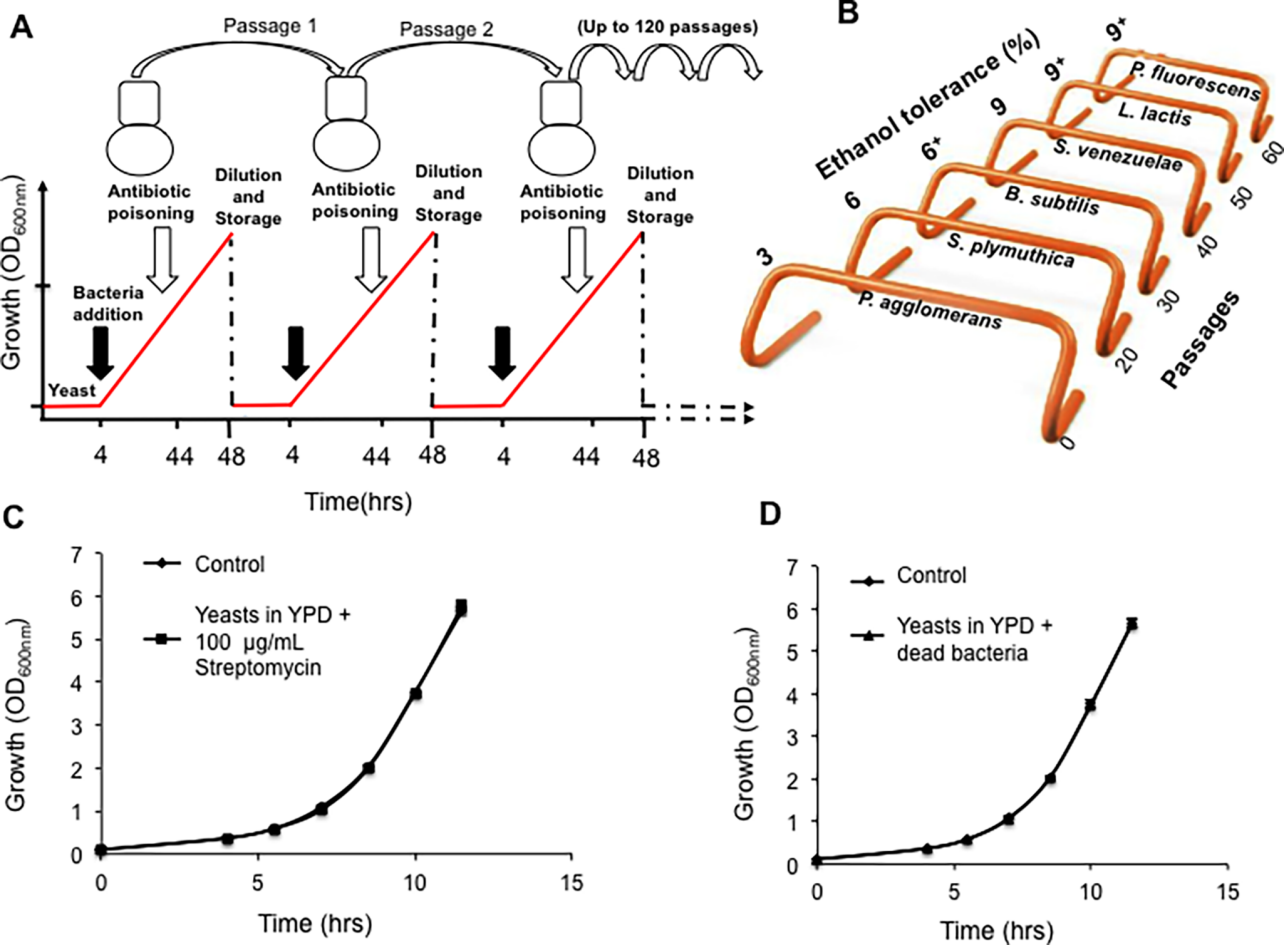
Experimental evolution scheme used to evolve yeasts. **A)** We used a modified serial dilution transfer method in which yeast and bacteria were co-cultured to compete only during the exponential phase to allow selection at maximum growth rates when resources were unlimited [46] by decreasing with time. Numbers 1 and 2 show successive passaging of cultures, whereas the red line indicates yeast biomass. Each passage entailed a 48-hour incubation period consisting of initial 4 hours of yeast adaptation, followed by 40 hours of bacteria-yeast competition and then 4 hours of antibiotic treatment to “kill off” bacterial survivors before a 500-fold dilution into fresh media. **B)** Bacterial “hurdles” scheme. The scheme shows the order of sequential exposure of yeasts to six bacteria species. *P*. *agglomerans* was used to added to growing yeasts for 20 passages and then another 20 passages with *S*. *plymuthica* and e.t.c. *Pseudomonas fluorescens* was the only species that was used for longer than 20 passages. Longitudinal samples were stored for analyses every 10 passages. **C)** Effects of streptomycin on growth of yeasts. The plot shows control yeasts grown in YPD (filled diamond) and yeasts grown in YPD supplemented with streptomycin (filled square). **D)** Effects of dead bacteria on growth of yeasts. The plot shows control yeasts grown in YPD (filled diamond) and yeasts grown in YPD together with dead bacteria (filled triangle). The figure shows that both Streptomycin and dead bacteria (heat-killed by incubating for 5 hours at 60°C) did not affect yeasts growth.

We set up a laboratory evolution experiment mimicking the natural environment characteristic of cross-kingdom competition for sugars in nature when fruits ripen leading to availability of excess sugars for a short period of time as previously reported [16, 20, 39]. In more detail, a total of 18 yeasts from the family *Saccharomycetaceae* covering a wide phylogenetic background spanning over 250 million years of evolutionary history [36] were cultured in the presence as well as in the absence of bacteria. We used a long-term serial dilution transfer method in aerobic shake flasks in YPD (2% glucose, 0.5% yeast extract and 1% peptone), at a pH of 6.2) in 250 mL baffled-bottom shake flasks at 25°C at 200 r.p.m in an Infors HT Ecotron shaker unit (Infors HT) as previously reported. An isogenic colony from each of these 18 yeasts was used to establish six independent evolution lines. Three flasks were inoculated with both yeast (at a concentration of 4 ± 0.05 log10 cfu/mL) and bacteria (at a concentration of 4 ± 0.05 log10 cfu/mL) whereas the other three flasks were inoculated with yeast only (serving as a triplicate of controls) (Fig 1). Both co-cultures and control cultures were passaged as shown in Fig 2A. For co-culture evolution, one species of bacteria was introduced at a time (for example, shown as red, yellow or green) in the order previously reported [16, 20] and shown in Fig 2B. After co-incubation for 44 hours, streptomycin (100 μg/mL) was added to kill “off” bacteria. We then withdrew 50 μL of the mixed cultures after 4 hours and transferred into fresh media. This procedure was repeated for several passages (Fig 2A). Longitudinal samples were stored after every 10 passages for analyses. Cultures from each of the six evolution lines were serially dilution plated out (10^−6^ cells/mL) and colonies from each plate were randomly picked and analyzed.

### Determination of order of bacterial hurdles

The order with which yeast encountered bacteria was determined by their reported ability to tolerate ethanol [20]. The first bacteria to be encountered were the most sensitive to ethanol whereas the last was the most ethanol tolerant. Tolerance of bacteria to ethanol was determined by growing them in LB (5 g/L yeast extract, 10 g/L peptone and 5 g/L NaCl adjusted to a pH of 7.4) supplemented with 3, 6, 9 and 10% (v/v) of ethanol as previously reported [20].

### Karyotyping of evolved lines

From each frozen sample stored after every 10 passages (number of generations vary due to different growth rates among species) we plated out cells (7.7 ± 0.1 log_10_ CFUs/mL) and randomly selected at least 3 independent colonies and determined their karyotypes using a CHEF Mapper XA PFGE apparatus (Bio-Rad). The preparation of chromosomal plugs for PFGE was done by standard methods as described before [16, 40]. Plugs were run using a multistate program as described previously [16].

### Phenotyping of evolved strains

The phenotype of the evolved strains was investigated using a high-throughput micro-cultivation instrument, Bioscreen C (Oy Growth Curves, Finland) that uses an automated system that measures turbidity of liquid cultures in a controlled environment, of up to 200 samples (2 x 100-well plates). In brief, we grew single colonies of each evolution line overnight in 96-well plates in YPD (0.5% yeast extract, 1% peptone, 2% glucose, pH 6.2). These overnight cultures were harvested halfway through exponential phase (OD_600nm_ < 0.8). 10 − 25 μL aliquots from the overnight cultures were used to inoculate multiwells to make a final volume of 200 μL of YPD medium (0.5% yeast extract, 1% peptone, 2% glucose, pH 6.2) to make an initial of OD_600nm_ of 0.05 to 0.1. Two wells were used as negative controls for background correction for each experimental run. Two positive controls inoculated with the ancestral strain were included. The Bioscreen C was used for incubation (with continuous shaking at 160 rpm) and monitoring the growth of the evolved strains at 25°C (temperature at which they were evolved). Turbidimetric readings were taken every 20 minutes for 168 hours and then the data were exported to excel for analyses. Only evolution lines that underwent genomic rearrangements were used in this study. Samples were run in duplicates and repeated twice.

## Results and discussion

### Yeast-bacteria competition methodology

Yeast-bacteria competition was hypothesized to be a probable trigger of genetic diversity among wild yeasts [32]. It is hypothesized that the emergence of angiosperms (glucose-rich flowering plants) over 125 million years ago [41], which created a sudden glut of fruit sugars [32], coincided with rewiring of carbon metabolism pathways among yeasts [32, 42]. A fierce competition for sugars between fruit inhabiting microbes, for example cross-kingdom competitors, bacteria and yeasts, could be the major evolutionary driving force accounting for genomic diversity we see in modern yeasts [16, 20, 32]. We therefore sought to reconstruct a primordial environment (an ecological battlefield) under laboratory conditions to investigate if competition between cross-kingdom competitors could have triggered the diversification of genomes evident in modern yeasts.

Experimental evolution is a very useful technique to answer such biological questions [3–5, 16, 43–46]. A modified serial dilution transfer experiment (Fig 2A) in which yeasts were co-cultured with bacteria to compete for the available sugars was chosen [16, 20]. YPD (0.5% yeast extract, 1% peptone, 2% glucose, pH 6.2) as a rich medium, was chosen to mimick wild fruits, a rich source of nutrients, a basis for competition. To ascertain that the possible outcome of competition will not differ among individual yeasts species or genera, 18 yeasts covering a wide phylogenetic background in the family *Saccharomycetaceae* were used (Table 1).

The next fundamental question we asked ourselves was: if bacteria are ubiquitous in nature, which bacteria should we choose to reconstruct an ecological battlefield that simulates the probable niche that reportedly coincided with diversification of modern yeasts characterised by aerobic ethanol production [32, 42]? We therefore chose bacteria whose niches are glucose-rich environments, vineyard and apple orchards, which are sympatric to many aerobic fermenting yeasts [18, 47, 48]. In addition, vineyard soils and grape tree leaves and trunks were chosen. However such environments have a complex microbial ecology [18], which is often too complex to mimic in laboratory conditions. We then isolated six microbes that grew well on glucose supplemented media and those that were resistant to ethanol, an ecosystem engineering metabolite produced by yeasts for niche defense purposes [31, 32, 49], as reported elsewhere [20]. For the practical setup of the experiment, two solutions would have been possible: The co-culturing of all bacteria species on Table 1 with yeasts in a single flasks was one way whereas the sequentially introduction of one bacteria species after the other was another. To create a less complex environment manageable under laboratory, each of the yeasts species (Table 1) was allowed to sequentially encounter each of the six bacterial species, one at a time, for 20 passages (Fig 2A and 2B). The order with which yeasts encountered bacteria was based on the sensitivity of bacteria to ethanol as previously reported [16, 20]. It follows our hypothesis that yeasts produce ethanol for niche defense purposes [32]. It is noteworthy that concentrations of ethanol in ripening and very ripened sugar-rich tropical palm fruits, range between 0.5% and 0.6% as reported by [50]. However, ethanol is very volatile to suggest that these ranges are far much lower than the actual possible values. We therefore chose to expose each yeast species to the least ethanol tolerant bacterium first whereas the most tolerant bacterium was the last one to be encountered. A higher ethanol tolerant bacterium is a bigger “hurdle” in the ability of yeast to oust it in a specified niche, as the yeast would require a higher ethanol titer to “kill off” the bacterium. Such a bigger “hurdle” would then translate to a stronger selection pressure towards a higher ethanol producing capacity. It is noteworthy that there is a gradient of strength of selection, from weakest to strongest. A weak selection pressure during the early stages of evolution served to allow the emergence of more genetically diverse variants, an attribute important for populations to explore a multiple of paths to adaptation as well as avoiding populations being driven to extinction as the solution associated with a strong selection pressure becomes inaccessible as reviewed in [5].

Noting that bacteria are fierce competitors in sugar abundant niches that could starve off yeasts, extensively discussed in [16], we were prompted to find an appropriate method to allow yeasts and bacteria to compete in a laboratory setup. We introduced bacteria into a flask with an already adapted yeast population (after lag phase) to avoid a rapid competitive exclusion before yeasts adapted to the growth media. The two cross-kingdom competitors were then allowed them to co-exist until late exponential phase to allow selection upon maximum growth rate. To exclude other possible selection pressures and outcomes that could arise due to stationary phases, such as nutrient-limitation, exhaustion and end product toxicity that could mask our bacterial competition selection pressure hypothesis set in the experiment [4, 5, 46], we transferred batch cultures before they reached the stationary phase. Thus this experimental set up explored the evolvability of yeasts during a competition with bacteria in the presence of initially excess glucose decreasing over the course of time. The decrease in nutrients is typical in nature when fruits ripen. Microbes consume nutrients to depletion before being dispersed to a fresh fruit. Similarly we couldn’t have done the experiments in a chemostat, because the mimicked ephemeral “ripened or rotting apple” microhabitat is not constant.

To avoid evolution of bacteria, an antibiotic drug (100 μg/mL of Streptomycin) was used to “kill off” the surviving bacteria before transferring into fresh media. Use of streptomycin was done to avoid evolution of bacteria, instead of yeasts, a scenario that could confound the set hypothesis. Survival of bacteria after antibiotic treatment (checked by plating antibiotic treated cultures on agar medium) could have led to yeasts being outcompeted during the next transfer step. Streptomycin was also added to the control experiments to avoid a discrepancy in the probable selection pressures between co-cultured yeasts and control yeasts if the negative effect of Streptomycin on yeast growth reported elsewhere holds [51]. In addition, an experiment was set up to investigate the effects of Streptomycin on yeasts. The results showed that the antibiotic drug did not lead to any reduction in growth capacity of yeasts (Fig 2C). We were also prompted to investigate the effects of dead bacteria (“ghosts” bacteria) on growth of yeasts. Fig 2D shows that the presence of dead bacteria did not have any unanticipated effects on growth of yeasts. It is characteristic of yeasts to undergo metabolic shifts even in a simple laboratory environment, as they tend to maximize their fitness and substrate utilization capacity [5], hence the justification of why we set the experimental evolution strategy as described above was mandatory.

### Library of experimentally evolved populations

All yeasts evolved in the presence or absence of bacteria were collected and stored after every 10 passages (8 − 14 generations per passage depending on species). It was clear that the presence of bacteria reduces the rate of growth among yeasts. This is evidenced by the discrepancy in number of passages reached by yeasts during the course of coevolution in the presence of bacteria (Fig 1) as compared to when yeasts were grown as pure cultures (Table 1). It should be noted that strains with a lower number of passages recorded had difficulties in growing in the presence of bacteria (Fig 1) whereas control strains were passaged more often accounting for higher numbers of passages tabulated. Strains with a lower number of passages recorded, were either driven to extinction during evolution or did not double in numbers to at least four times the initial concentrations per passage. We set the number of doubling times to be at least four because all the control strains doubled between 8 − 14 times per passage when grown as pure cultures. When the experimental populations were driven to extinction, based on quantification using microscopy, frozen stocks from the preceding passage were revived and used as the seed culture. The constant revisiting of the frozen stocks meant that the number of passages would not tally with those of the control lines as well as those cultures that did not suffer the same fate. All control lines were passaged for 120 times except *C*. *glabrata*, which was passaged for 130 passages).

### Genome reorganization

**Fig 3:**
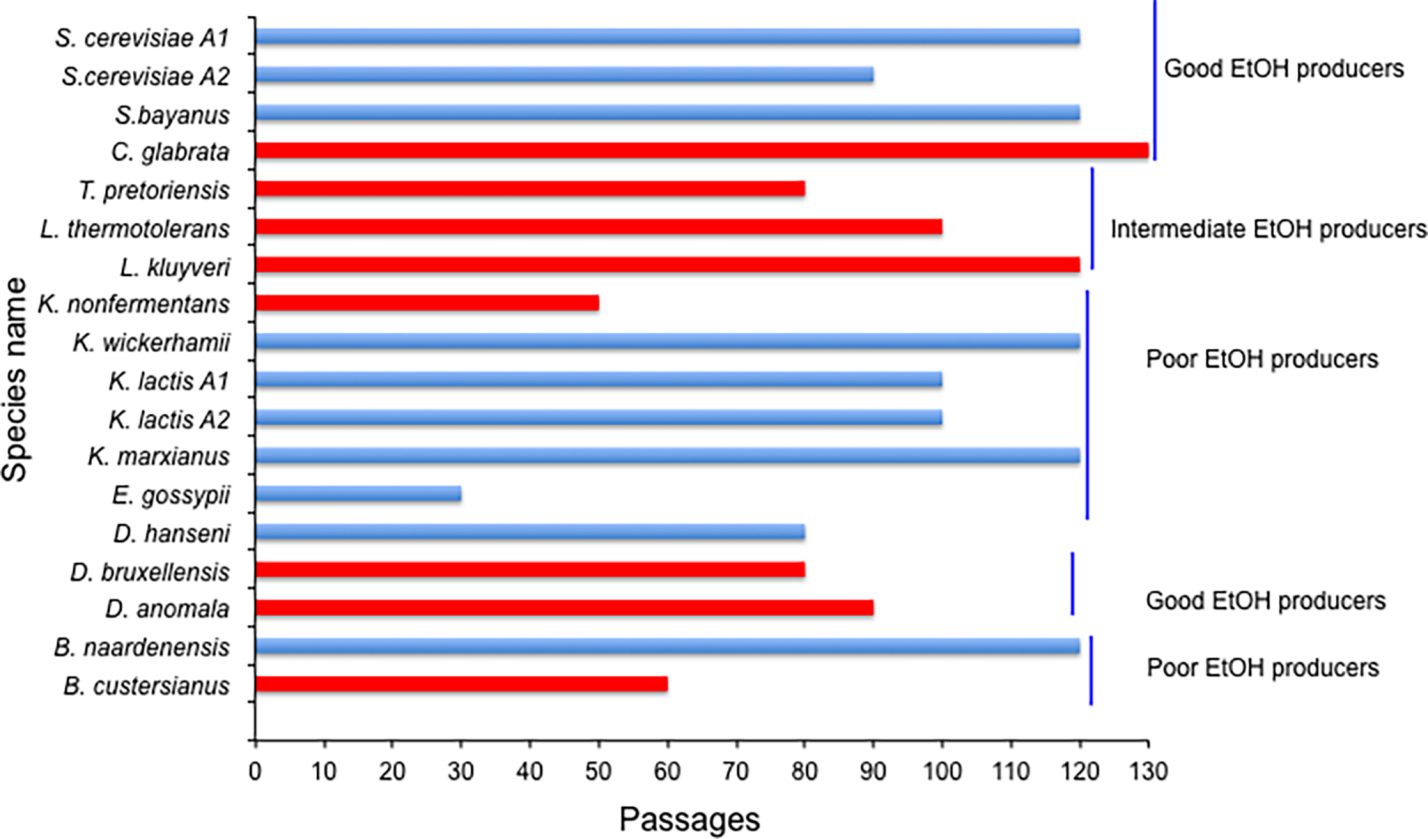
Long-term yeast-bacteria experimental evolution. A total of 18 yeasts covering over 250 million years of evolutionary history (*S*. *cerevisiae* A1 and A2 (2 strains), *S*. *eubayanus*, *C*. *glabrata*, *T*. *pretoriensis*, *L*. *thermotolerans*, *L*. *kluyveri*, *K*. *non-fermentans*, *K*. *wickerhamii*, *K*. *lactis* A1 and A2 (2 strains), *K*. *marxianus*, *E*. *gossypii*, *D*. *hansenii*, *D*. *bruxellensis*, *D*. *anomala*, *B*. *naardenensis*, and *B*. *custersianus*) [36] evolved in this study are shown. Yeasts are phylogenetically ordered such that species at the bottom are the least related to *S*. *cerevisiae* [60]. Red bars represent yeasts that underwent genomic rearrangements whereas blue bars represent those that did not. *Poor, intermediate and good ethanol producers (i.e. ethanol yield of 0.1 ± 0.1g, 0.25 ± 0.05g and 0.37 ± 0.06g of ethanol per gram of glucose consumed in aerobic batch fermentation respectively as reported elsewhere) [61] are all represented in the *Saccharomyces* and *non-Saccharomyces* yeasts. Species with a lower number of passages had difficulties in growing in the presence bacteria as described in the main text.

**Fig 4:**
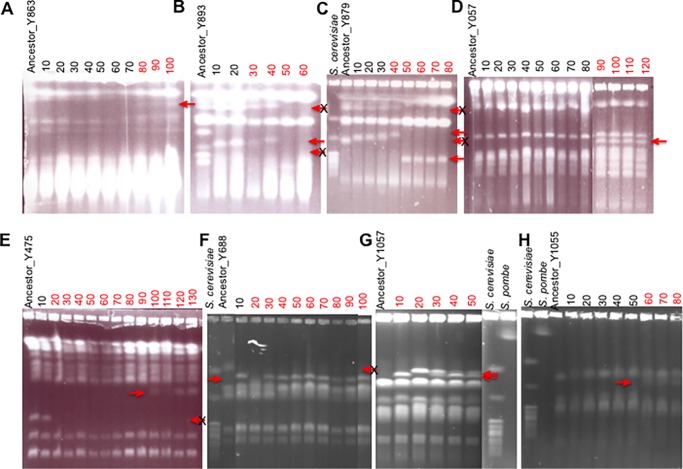
Evolutionary trajectories based on electrophoretic karyotypes. The figure shows chromosomal bands separated by PFGE using a CHEF Mapper XA PFGE apparatus (Bio-Rad). To determine karyotypes, overnight cultures from each of the frozen samples stored after every 10 passages were used. Preparation of chromosomal plugs for PFGE was done by standard methods as reported [40]. Plugs were run using a multistate program for 110 h (Block 1, 25 h at 1.5 V/cm with a 2, 700 s pulse time and an angle of 53°; Block 2, 25 h at 1.5V/cm with a 2, 200s pulse time and an angle of 60°; Block 3, 30 h at 2 V/cm with a 1.500 s pulse time and an angle of 60°; and Block 4, 30 h at 2.5 V/cm with a 500-s pulse time and an angle of 60°) at a constant temperature (14°C) [52]. The gels were stained with ethidium bromide before photographing. Most ancestral strains used in this study are completely sequenced to be used as standards to estimate the sizes of novel bands based electrophoretic migration distance regression curve. In some cases, *S*. *cerevisiae* (S288c) and *Schizosaccharomyces pombe* (SJA148) were used as standards. Strains that underwent a rearrangemnt event are labelled in red. Bands that were not found on the respective ancestral lane are shown by a red arrow whereas those that were lost are shown by a cancelled red arrow. *D*. *anomala* (Y863), *B*. *custercianus* (Y893), *D*. *bruxellensis* (Y879), *L*. *kluyveri* (Y057) [16], *C*. *glabrata* (Y475), *L*. *thermotolerans* (Y688), *K*. *nonfermentans* (Y1057) and *T*. *pretoriensis* (Y1055).

We did not observe any changes in karyotypes in yeasts grown in the absence of bacteria (data not shown) whereas we detected large-scale genomic rearrangements in 45% of the total number of yeasts (8 out 18 yeasts under investigation) grown under a bacterial challenge: *D*. *anomala* (Y863), *B*. *custercianus* (Y893), *D*. *bruxellensis* (Y879), *L*. *kluyveri* (Y057) [16], *C*. *glabrata* (Y475), *L*. *thermotolerans* (Y688), *K*. *nonfermentans* (Y1057) and *T*. *pretoriensis* (Y1055*)* (specific strains marked in red) (Figs 3 and 4) (see also Table 2).

**Table 2:** Long-term evolution experiment. The table lists culture collection identities and the number of passages they were evolved (in the presence of a bacterial selection pressure). All controls were passaged for 120 times except *C*. *glabrata*, which was passaged for 130 passages). Strains that underwent large-scale genomic rearrangements were derived from species in bold.

Origin	Lund culture collection number	Species name	^a^Control passages	^b^Co-culture passages	^c^Evolution lines with rearrangements
**Yeasts**					
CBS 8340	Y706	*Saccharomyces cerevisiae* A1	120	120	0
CBS 7413	Y1714	*Saccharomyces cerevisiae* A2	120	90	0
CBS 12357	Y1693	*Saccharomyces eubayanus*	120	120	0
**CBS 138**	**Y475**	***Candida glabrata***	**130**	**130**	**2**
**CBS 2926**	**Y1055**	***Torulaspora pretoriensis***	**120**	**80**	**2**
**CBS 6340**	**Y688**	***Lachancea thermotolerans***	**120**	**100**	**2**
**^d^CBS 3082**	**Y057**	***Lachancea kluyveri***	**120**	**120**	**2**
**CBS 8778**	**Y1057**	***Kluyveromyces nonfermentans***	**120**	**50**	**3**
CBS 2745	Y113	*Kluyveromyces wickerhamii*	120	120	0
CBS 2359	Y707	*Kluyveromyces lactis* A1	120	100	0
CBS 2359	Y1376	*Kluyveromyces lactis* A2	120	100	0
CBS 712	Y1058	*Kluyveromyces marxianus*	120	120	0
CBS 109.51	Y1001	*Eremothecium gossypii*	120	30	0
CBS 6920	Y1399	*Debaryomyces hansenii*	120	80	0
CBS 2499	**Y879**	***Dekkera bruxellensis***	**120**	**80**	**3**
CBS 77	**Y863**	***Dekkera anomala***	**120**	**100**	**2**
CBS 6116	Y919	*Brettanomyces naardenensis*	120	120	0
**CBS 4805**	**Y893**	***Brettanomyces custersianus***	**120**	**60**	**2**

^a^Number of passages in which yeasts were evolved in the absence of bacteria, i.e. controls

^b^Number of passages in which yeasts were evolved in the presence of bacteria. For example, *C*. *glabrata* was passaged for 120 times

^c^Lines which underwent rearrangements out of 3 evolution lines grown in the presence of bacteria.

^d^Genomic rearrangements reported elsewhere [16].

In six out of the eight cases in which genomic rearrangements were observed, at least two out of three evolution lines in each species underwent genomic rearrangements (Table 2). We noted only two cases where all three evolution lines grown under a bacterial selection pressure underwent genomic rearrangements i.e. *D*. *bruxellensis* (Y879) and *Kluyveromyces nonfermentans* (Y1057) (Table 1). Genomic rearrangements appeared in last evolved passages derived from *D*. *anomala* (Y863), *T*. *pretoriensis* (Y1055) and *L*. *kluyveri* (Y057) (Fig 4A and 4D) whereas in other cases the rearrangements appeared in early passages during the evolution process. Up to four sampled passages derived from *B*. *custercianus* (Y893) and *L*. *kluyveri* (Y057) exhibited extra-chromosomal bands (Fig 4B and 4D).

All sampled passages derived from *C*. *glabrata* (Y475) and *L*. *thermotolerans* (Y688) except the first strains isolated after 10 passages gained a chromosome (Fig 2E and 2F). Chromosomal rearrangements were also evidenced by samples taken from *K*. *nonfermentans* (Y1057). In addition, we also noted that some passages derived from *B*. *custercianus* (Y893), *D*. *bruxellensis* (Y879), *C*. *glabrata* (Y475), and *L*. *thermotolerans* (Y688) lost either one or two chromosomes as evident in the disappearance of chromosomal bands that were present in the ancestral strain lanes (Fig 4B, 4C, 4E and 4F).

It is noteworthy that karyotype variability within and between species is a striking feature of *Saccharomyces* yeasts [40].The feature is also common among their distant relatives that diverged about 200 million years ago, *Dekkera/Brettanomyces* [52–54]. These yeasts either as natural isolates, clinical, laboratory or industrial yeast strains exhibit a striking karyotype variability [43, 44, 55]. Ahmad *et al*., 2013 [55] reported that reshuffling and rearrangement of the genomes is a proven mechanism that increases virulence among clinical *C*. *glabrata* strains. On the other hand in contrast to most laboratory strains, there is a huge discrepancy of karyotypes of yeasts used in the industry suggesting that genetic reconfigurations by yeasts are a successful strategy for survival and reproduction in response to harsh environmental conditions [56, 57].

Whole genome sequence of genomic rearrangements observed in *L*. *kluyveri* reported, elsewhere revealed a duplication and translocation event involving a 261-kb fragment [16]. This fragment harboured genes involved in carbon metabolism and stress tolerance such osmotic stress, ethanol as well as DNA replication stress. The results suggest that large-scale genomic rearrangements are a genetic reservoir for natural selection to act upon and play a major role in yeasts evolvability in nature.

### Phenotypic changes due to genomic restructuring

**Fig 5:**
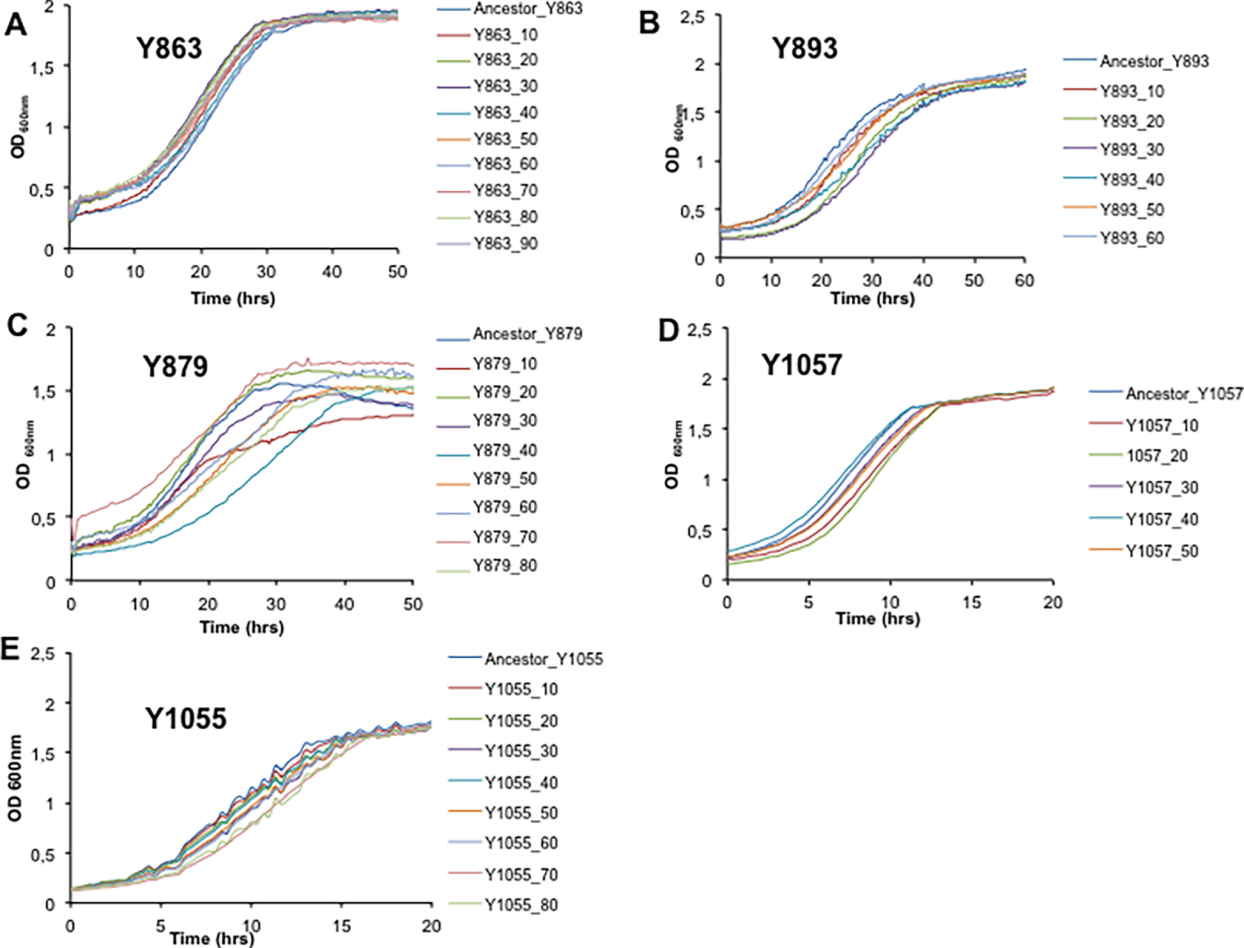
Phenotyping of the evolved strains. Strain phenotyping was performed using a high-throughput micro-cultivation instrument, Bioscreen C (Oy Growth Curves Ab Ltd, Helsinki, Finland). Turbidimetric readings were recorded every 20 minutes for 168 hours. Strains were grown at 25°C in rich medium, YPD (0.5% yeast extract, 1% peptone, 2% glucose, pH 6.2). The plots show average growth data from duplicates experiments. The experiments were carried out for 168 hours although we only show the lag and exponential phases of growth on these plots. **A)** *D*. *anomala* (Y863), **B)** *B*. *custercianus* (Y893), **C)** *D*. *bruxellensis* (Y879), **D)** *K*. *nonfermentans* (Y1057), and **E)** *T*. *pretoriensis* (Y1055). *C*. *glabrata* could not be tested using the same facilities as the species is an opportunistic pathogen. Phenotypes of *L*. *thermotolerans* (Y688) are not reported in this work, whereas *L*. *kluyveri* (Y057) has been comprehensively described elsewhere [16].

We examined a fundamental characteristic of the evolving populations, namely phenotypic changes in growth rate relative to that of their ancestral strains. Fig 5 shows an example of average growth profiles of the derived strains obtained using, Bioscreen C (Oy Growth Curves, Finland), a high-resolution micro-cultivation approach [58]. These results show that the derived strains are also phenotypically divergent from their parental strains.

**Fig 6:**
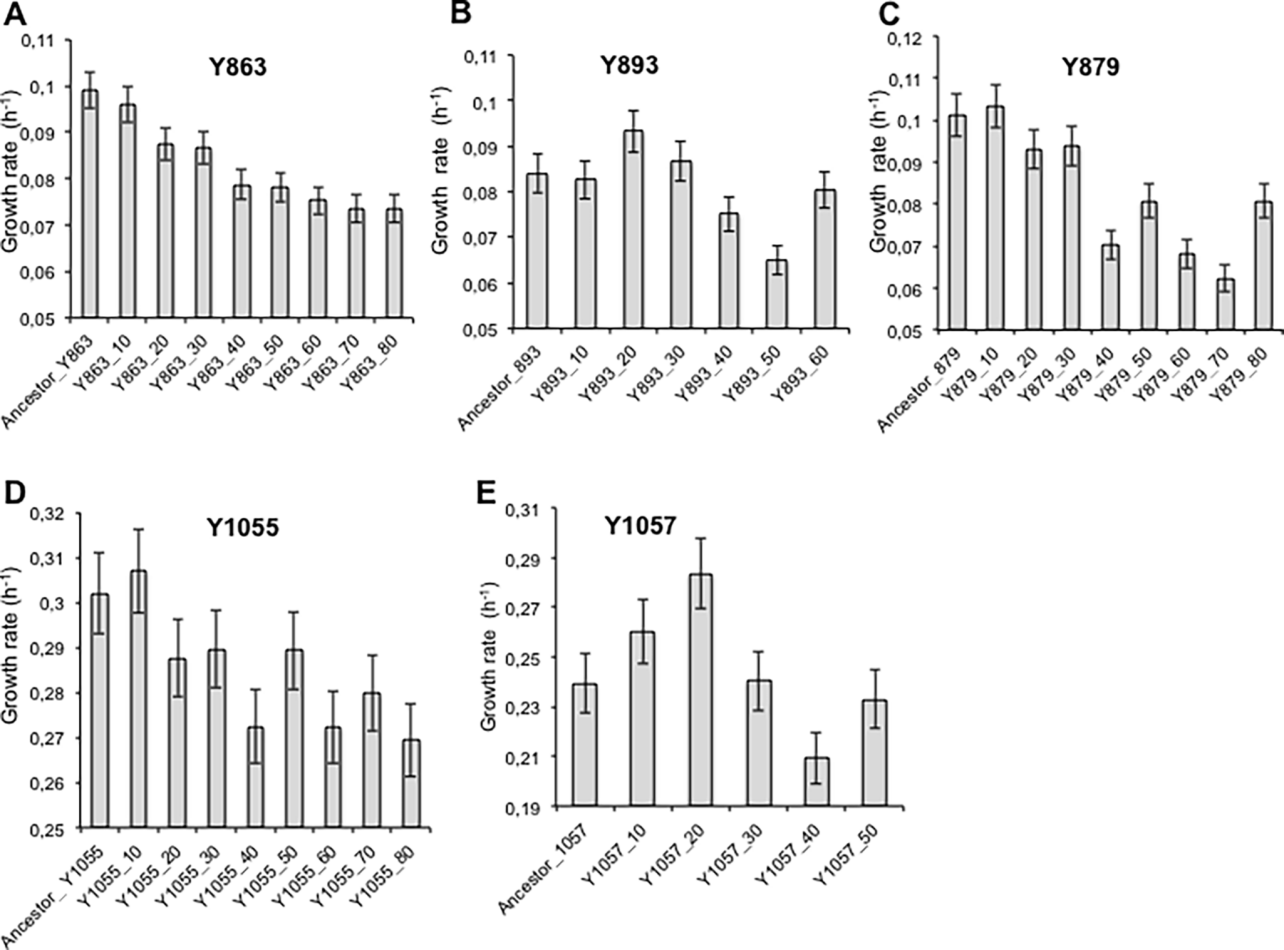
Growth rates variation among the evolved strains. Growth rates were calculated from the data in Fig 3. The growth rates were calculated from turbidimetric readings acquired at OD_600nm_ during the exponential phase (S1 File). A linear regression of log of readings was done to calculate the growth rates using Microsoft Excel. The data shown is an average of duplicate assays. **A)** *D*. *anomala* (Y863), **B)** *B*. *custercianus* (Y893), **C)** *D*. *bruxellensis* (Y879), **D)** *T*. *pretoriensis* (Y1055) and **E)** *K*. *nonfermentans* (Y1057). *C*. *glabrata* could not be tested using the same facilities as the species is an opportunistic pathogen. Phenotypes of *L*. *thermotolerans* (Y688) are not reported in this work whereas *L*. *kluyveri* (Y057) has been comprehensively described elsewhere [16].

In general there was a decrease in growth rates in strains sampled along the timeline of the experiment derived from *D*. *anomala*, *D*. *bruxellensis and K*. *nonfermentans* (Fig 6). In contrast the growth rates of strains evolved from *B*. *custercianus*, *K*. *nonfermentans* and *T*. *pretoriensis* suggest that genome reorganization may also retard the growth abilities of the resultant strains as growth rates go up and down in some cases, and decline in others. This could be explained by changes in the selection pressure when swapping one bacterium for another. As reported previously, there was an increase in growth rates in strains derived from *L*. *kluyveri* with increasing exposure to bacteria [20]. When functional characterization of the derived genetic variants was done, for example, the effects of genome adaptations under different environmental conditions, we observed that after 120 passages the yeast rewired its carbon metabolism to outcompete bacteria, as characterised by their evolved antibacterial activity, which was not evident in the ancestral strain. These strains were also characterised by a doubling in ethanol production, as well as ability to utilize a higher number of carbon sources as well stress tolerance as compared to their ancestor [20].

These results suggest that genomic reorganization in yeast is an attribute exploited by yeasts to increase their chances of survival in a wide range of niches.

### Exploitation of yeasts’ ability to restructure genomes in modern biotechnological processes

Baking, brewing and winemaking are examples of applications of yeasts that constitute an enormous segment of the food market. There is an increasing demand by customers for new flavors or by producers for more robust (reproducible) phenotypes. Another example is the use of yeasts for biofuel production, where an increased thermotolerance is desirable. Most studies have focused on scouting for yeasts with attractive phenotypic attributes in pristine environments. Although an immense biodiversity exist, yeasts do not necessarily possess phenotypic traits that are directly transferrable to food, biotechnology or industrial applications [45]. It is therefore important to combine the natural biodiversity and strain development strategies to generate and optimize yeast strains with traits suitable for specific conditions (reviewed in [2, 59]). Here we report a multitude of genetic variants from our experimental evolution approach highlighting how a cross-kingdom selection may be a useful technique to develop strains of industrial importance.

With the rise of high throughput next-generation sequencing platforms, it is becoming increasingly easier to track evolutionary trajectories based on global genomic and transcriptional changes in comparison to their ancestral founding genotypes. Such approaches allow us to understand genetic pathways important for selection of improved phenotypes [2, 3, 6].

## Conclusions

This study further increases our understanding of ecological processes that lead to evolution of extreme adaptive phenotypes in the context of interspecies communities. At the same time, our collection of modified strains reveals how a novel two-species cross-kingdom competition strategy may be exploited to reprogram yeast genomes and subsequent metabolic networks for industrial applications.

## Supporting information

S1 File Bioscreen results. The file contains average OD_600nm_ readings of strains phenotyped using the Bioscreen C system. *D*. *anomala* (Y863), *B*. *custercianus* (Y893), *D*. *bruxellensis* (Y879), *T*. *pretoriensis* (Y1055), *K*. *nonfermentans* (Y1057), and *L*. *thermotolerans* (Y688) data is shown. Data compilation for 2-hour intervals was extracted and used to calculate the growth rates in Fig 6. Yellow highlighted data points represent exact points used to calculate the growth rates. Strains passaged for a different number of times are show. For example, Y863_10 means a resultant strain originating from passaging the ancestral strain, Y863, for 10 times was analysed.
(XLSX)
